# SARS-CoV-2 Spike protein alters microglial purinergic signaling

**DOI:** 10.3389/fimmu.2023.1158460

**Published:** 2023-04-11

**Authors:** Vinícius Santos Alves, Stephanie Alexia Cristina Silva Santos, Raíssa Leite-Aguiar, Elaine Paiva-Pereira, Renata Rodrigues dos Reis, Mariana L. Calazans, Gabriel Gripp Fernandes, Leticia Silva Antônio, Emanuelle V. de Lima, Eleonora Kurtenbach, Jerson Lima Silva, Fabricia Lima Fontes-Dantas, Giselle Fazzioni Passos, Cláudia Pinto Figueiredo, Robson Coutinho-Silva, Luiz Eduardo Baggio Savio

**Affiliations:** ^1^ Instituto de Biofísica Carlos Chagas Filho, Universidade Federal do Rio de Janeiro, Rio de Janeiro, Brazil; ^2^ Faculdade de Farmácia, Universidade Federal do Rio de Janeiro, Rio de Janeiro, RJ, Brazil; ^3^ Instituto de Bioquímica Médica Leopoldo de Meis, Universidade Federal do Rio de Janeiro, Rio de Janeiro, RJ, Brazil; ^4^ Departamento de Farmacologia e Psicobiologia, Instituto de Biologia Roberto Alcântara Gomes Institute Biology (IBRAG), Universidade Estadual do Rio de Janeiro (UERJ), Rio de Janeiro, Brazil

**Keywords:** SARS-CoV-2 spike protein, COVID-19, ectonucleotidase, P2 receptors, CD39, neuroinflammation, microglia

## Abstract

Despite long-term sequelae of COVID-19 are emerging as a substantial public health concern, the mechanism underlying these processes still unclear. Evidence demonstrates that SARS-CoV-2 Spike protein can reach different brain regions, irrespective of viral brain replication resulting in activation of pattern recognition receptors (PRRs) and neuroinflammation. Considering that microglia dysfunction, which is regulated by a whole array of purinergic receptors, may be a central event in COVID-19 neuropathology, we investigated the impact of SARS-CoV-2 Spike protein on microglial purinergic signaling. Here, we demonstrate that cultured microglial cells (BV2 line) exposed to Spike protein induce ATP secretion and upregulation of P2Y_6_, P2Y_12_, NTPDase2 and NTPDase3 transcripts. Also, immunocytochemistry analysis shows that spike protein increases the expression of P2X7, P2Y_1_, P2Y_6_, and P2Y_12_ in BV2 cells. Additional, hippocampal tissue of Spike infused animals (6,5ug/site, i.c.v.) presents increased mRNA levels of P2X7, P2Y_1_, P2Y_6_, P2Y_12_, NTPDase1, and NTPDase2. Immunohistochemistry experiments confirmed high expression of the P2X7 receptor in microglial cells in CA3/DG hippocampal regions after spike infusion. These findings suggest that SARS-CoV-2 Spike protein modulates microglial purinergic signaling and opens new avenues for investigating the potential of purinergic receptors to mitigate COVID-19 consequences.

## 1 Introduction

COVID-19, the disease caused by the severe acute respiratory syndrome coronavirus 2 (SARS-CoV-2), is associated with variable outcomes. Beyond the respiratory complications produced by the viral infection, a range of neurological complications may occur in the acute phase of COVID-19, including confusion, strokes, and neuromuscular disorders. Additionally, about 35% of infected patients with different ages and severity of the disease experience persistent attention and memory deficits, called as Long COVID (1, 2). Several studies have proposed that neurological dysfunctions seen in COVID-19 may be due to different mechanisms, including ischemic and inflammatory events. Moreover, SARS-CoV-2 may lead to pro-inflammatory and cytotoxic scenarios in the central nervous system regardless of local viral replication, induced only by its presence in the tissues (3). Nevertheless, whether the brain presence of SARS-CoV-2 viral particles and/or its products is a crucial event for the development of cognitive impairment in post-COVID patients remains unknown.

Microglia are the most prominent immune and cytokine-producing cells in the brain that respond to pathogen-associated molecular patterns (PAMPs) and damage-associated molecular patterns (DAMPs) to drive innate immune responses and neuroinflammation. Viral infections modulate microglial functions resulting in pathological synaptic remodeling, which culminates in altered cognition and behavior (4, 5). Matschke and colleagues (6) showed that post-mortem analysis of brain tissue from COVID-19 patients presented intense microgliosis and neuroinflammatory response. Microglial activation in SARS-CoV-2 infections has also been demonstrated *in vivo* and *in vitro* models (7–10). Recently, we and others demonstrated that SARS-CoV-2 Spike protein S1 subunit appears to act as a pathogen-associated molecular pattern through TLR4, activating microglia and inducing secretion of proinflammatory mediators (8, 10–13). Our group also demonstrated that mice brain infusion of Spike protein induces late cognitive dysfunction, recapitulating post-COVID syndrome, through microglia-dependent engulfment of synapses (13).

Purinergic system is one of the fundamental signaling systems that establish microglial behavior in a wide spectrum of conditions (14). Purinergic signaling is a preserved pathway across several species stimulated by nucleotides and nucleosides such as ATP and ADP and involves purinergic receptors and purinergic enzymes called ectonucleotidases. Purinergic receptors differ in their ligands and specificity. They are classified into three types: P1, mobilized by adenosine (composed of four subtypes: A_1_, A_2A_, A_2B_, and A_3_), and the subtypes that are mobilized by nucleotides: P2X(1–7) and P2Y_(1,2,4,6,11–14)_ receptors (14–16).

P2X7, P2X4, P2Y_4_, P2Y_6_, P2Y_12_, A_2A_, and A_3_ are the primary purinergic receptors expressed by microglial cells. These receptors are associated with neurodegenerative events and may act as protective or degenerative mediators, depending on the disease and its progression (15, 17, 18). Many classes of purinergic receptors (P2Y_12_, P2Y_6_, P2Y_4_, P2X4, P2X7, A_2A_, and A_3_) can influence microglial behavior. Of note, P2X7 receptor is critical for microglial activation and secretion of proinflammatory cytokines, such as IL-1β, IL-18, and IL-6 (19, 20). P2Y_12_ and A_3_ receptors are primary participants after neuronal damage, modulating microglial activation and extension process (18). Furthermore, P2Y_12_ acts on microglia migration (18). Nevertheless, no studies have addressed the effects of SARS-CoV2 on microglial purinergic signaling. Therefore, to further investigate the impact of microgliosis in SARS-CoV-2 pathology, we investigated the effect of Spike protein on microglial purinergic signaling. Our findings suggest that SARS-CoV-2 Spike protein, independently of viral replication, is able to modify the expression of purinergic receptors involved in neuroinflammation, highlighting the relevance of these targets to managing long COVID symptoms.

## 2 Material and methods

### 2.1 Cell culture

BV-2 cells were cultured in RPMI-1640 medium (Sigma-Aldrich, MO, USA) supplemented with 10% fetal bovine serum (Sigma-Aldrich, MO, USA), antibiotics (100 IU penicillin/mL and 100 mg streptomycin/mL; Gibco^®^). Cells were tested for mycoplasma contamination.

### 2.2 Animals

In this study, we used 8–12-week-old male Swiss mice. Animals were housed in groups of five per cage with free access to food and water, a 12-h light/dark cycle, and controlled temperature and humidity. All procedures followed the Principles of Laboratory Animal Care (US National Institutes of Health) and were approved by the Institutional Animal Care and Use Committee of the Federal University of Rio de Janeiro (protocol number: 068/2).

### 2.3 SARS−CoV−2 spike protein *in vitro* and *in vivo* stimulation

The trimeric spike protein (1–1208aa) of SARS-CoV-2 was purified according to Cunha et al. (21) and obtained from the Cell Culture Engineering Laboratory of COPPE/UFRJ. Spike protein purity and antigenicity have already been confirmed in previous studies (21, 22). For *in vitro* experiments, BV-2 cells were plated in 6- or 24-well tissue culture plates (TPP AG, Switzerland) at 1 x 10^6^ or 2 x 10^5^ cells per well. Cells were left untreated or stimulated with 0.5 or 1 µg/mL SARS−CoV−2 spike protein. After spike protein stimulation, cell supernatants were tested for endotoxin contamination using the Pierce™ Chromogenic Endotoxin Quant Kit (Thermo Scientific, NJ, USA). Endotoxin levels were < 0.05 EU/mL.

To evaluate the effects of spike protein *in vivo*, adult male Swiss mice were anesthetized with 2.5% isoflurane (Cristália; São Paulo, Brazil) using a vaporizer system (Norwell, MA) and 6.5 µg spike protein or vehicle (saline) were slowly infused using a Hamilton syringe into the lateral ventricle (ICV). After 45 days of SARS−CoV−2 spike protein ICV infusion, animals were euthanized, and hippocampal tissues of each group were quickly separated and stored in liquid nitrogen before use.

### 2.4 ATP release assay

BV-2 cells were plated in 6-well tissue culture plates (TPP AG, Switzerland) at 1 x 10^6^ cells per well. Cells were left untreated or stimulated with 0.5 or 1 µg/mL SARS−CoV−2 spike protein for 2 h. The measurements of eATP from the culture supernatant were performed using the ATP determination kit (Life^®^ Probes; #A22066) by real-time luminometry, according to the manufacturer’s instructions. The luminescence of samples plated onto black 96-well plates was read in a SpectraMax^®^M5/M5e Multimode Plate Reader (Molecular Devices), and results were expressed as picomoles of ATP.

### 2.5 RNA isolation and real-time quantitative PCR

The total RNA was isolated using the ReliaPrep™ RNA Miniprep Systems kit (Promega Corporation, WI, USA) according to the manufacturer’s instructions. RNA samples were quantified, and the purity was assessed using a Nanodrop Lite spectrophotometer (Thermo Scientific, NJ, USA). The synthesis of cDNA was performed using 1 µg of total RNA using the High-Capacity Reverse Transcription Kit with RNase Inhibitor (Thermo Fisher Scientific, NJ, USA) according to the manufacturer’s instructions in a Master Cycler Gradient thermocycler (Eppendorf, Hamburg, Germany).

The real-time quantitative PCR reactions (RT-qPCR) were performed using the GoTaq^®^ qPCR Master Mix (Promega Corporation, WI, USA) in a QuantStudio 1 Real-Time PCR System (Thermo Scientific, NJ, USA). The reactions were performed in a final volume of 10 μl, using 2 μl of diluted cDNA (1:10) and 300 nM of each primer (5’-3’): *P2rx4* forward AGACGGACCAGTGATGCCTAAC and reverse TGGAGTGGAGACCGAGTGAGA; *P2rx7* forward AATCGGTGTGTTTCCTTTGG and reverse CCGGGTGACTTTGTTTGTCT; *P2ry1* forward GACTGACTGGATCTTCGGGGA and reverse CCACCACAATGAGCCACACC; *P2ry2* forward TGACGACTCAAGACGGACAG and reverse GTCCCCTACAGCTCCCCTAC; *P2ry4* forward ACTGGCTTCTGCAAGTTCGT and reverse AGGCAGCCAGCTACTACCAA; *P2ry6* forward TGCTGCTTGGGTAGTGTGTGG and reverse GTAAGGCTATGAAGGGCAGC; *P2ry12* forward CACAGAGGGCTTTGGGAACTTA and reverse GATTCAGCAGAAGCAGGACCA; *Entpd1* forward AGCTGCCCCTTATGGAAGAT and reverse TCAGTCCCACAGCAATCAAA; *Entpd2* forward TTCCTGGGATGTCAGGTCT and reverse GTCTCTGGTGCTTGCCTTTC; *Entpd3* forward ACCTGTCCCGTGCTTAAATG and reverse AGACAGAGTGAAGCCCCTGA; *Nt5e* forward CAGGAAATCCACCTTCCAAA and reverse AACCTTCAGGTAGCCCAGGT; *Actb* forward TATGCCAACACAGTGCTGTCTGG and reverse TACTCCTGCTTGCTGATCCACAT. The relative cDNA expression was calculated using the comparative cycle threshold method. The β-actin gene (*Actb*) was used as an endogenous control. The results were expressed as relative expression of the gene of interest/*Actb*.

### 2.6 Ectonucleotidase activity assays

Ecto-nucleoside triphosphate diphosphohydrolases (E-NTPDases) activities were estimated in a reaction medium consisting of a 20 mM Hepes buffer (pH 7.5) containing 1 mM CaCl_2_, 120 mM NaCl, 5 mM KCl, 60 mM glucose, 1 mM sodium azide and 0.1% mM albumin (all reagents from Sigma-Aldrich, MO, USA). BV2 cells (2 x 10^5^) were used in a final volume of 200 µl of reaction medium, and enzymatic reactions were started by the addition of ATP for a final concentration of 2 mM, followed by incubation for 30 minutes at 37 °C. Reactions were stopped by the addition of 200 µl of 10% trichloroacetic acid (TCA) (Sigma-Aldrich, MO, USA). Incubation times, protein concentrations, reaction mixtures, and substrate concentrations were chosen based on a previous study (23, 24). The amount of inorganic phosphate (Pi) released was measured using the colorimetric method described by Chan et al. (25). Controls to correct for non-enzymatic Pi in the samples were performed. All reactions were performed in triplicates, and enzyme activities were expressed in nmol Pi released per minute per number of cells.

### 2.7 Immunocytochemistry and cell microscopy analysis

BV-2 cells were plated in 24-well plates at a density of 2 x 10^5^ cells per well and stimulated with 1 µg/mL SARS−CoV−2 spike protein for 24 h. After stimulation, cells were fixed with 4% paraformaldehyde and 4% sucrose for 15 min at room temperature, permeabilized with 0.5% Triton X-100 for 30 minutes (except for cells were stained with extracellular anti-P2X7 antibody), and then blocked with 10% horse serum and 1% bovine serum albumin (BSA) in phosphate-buffered saline (PBS) for 30 min at room temperature. Samples were then incubated overnight with the following primary antibodies diluted in 0.1% BSA in PBS: rabbit anti-P2X7 #APR-008 (1:100), rabbit anti-P2Y_1_ #APR-009 (1:100), rabbit anti-P2Y_4_ #APR-006 (1:300), rabbit anti-P2Y_6_ #APR-011 (1:500), rabbit anti-P2Y_12_ #APR-012 (1:50) (Alomone Labs, Jerusalem, Israel), goat anti-CD39 #AF4398 (1:200) (R&D Systems, Minneapolis, MN), rabbit ENTPD2/CD39L1 # BS-11515R (1:100) (Thermo Fisher Scientific, NJ, USA). Cells were then washed and incubated at room temperature for 1 h with the secondary antibody (diluted to 1:300, in 0.1% BSA in PBS): anti-rabbit IgG (H+L)-Alexa Fluor^®^ 594 (Thermo Fisher Scientific, NJ, USA). Samples were stained with Hoescht nuclear dye (1:10,000) (Life Technologies, Eugene, OR) and then mounted and examined in a fluorescence microscope Zeiss AxioVert 200M. Three-dimensional images (Z-stack) were obtained on a Spinning Disk Confocal Microscope ZEISS Cell Observer SD (Peabody, MA, USA). Mean fluorescence intensity was measured using Zen Lite Blue software (Carl Zeiss). For this quantification, the background was initially subtracted, and the region of interest was selected in individual cells using the software’s freehand selection tool to calculate the mean fluorescence intensity in the presence of different treatments. The software calculated these results based on the intensity of the corresponding pixels. The experiments were performed in triplicate, and 70–90 cells per field were evaluated in ten fields per coverslip.

### 2.8 Immunofluorescence assay and analysis

Slides containing the hippocampal formation were deparaffinized, and antigen retrieval was carried out by incubation in citrate buffer solution (pH 6.0) at 95 °C for 40 min. Then, samples were incubated with blocking buffer (PBS containing 0.025% Triton, 3% BSA, and 5% normal goat serum) for 2 h. Slides were then incubated overnight with primary antibody rabbit anti-Iba-1 (FUJIFILM Wako Pure Chemicals Corporation, Osaka, Japan; #019-19741). On the following day, samples were washed and incubated at room temperature for 1 h with the secondary antibody (diluted to 1:300, in 0.1% BSA in PBS): anti-rabbit IgG (H+L)-Alexa Fluor^®^ 546 (Thermo Fisher Scientific, NJ, USA). For the second staining, samples were washed, blocked again, and incubated overnight with primary rabbit Anti-P2X7 (Alomone Labs, Jerusalem, Israel; #APR-008). On the third day, samples were washed and incubated at room temperature for 1 h with the secondary antibody (diluted to 1:300, in 0.1% BSA in PBS): anti-rabbit IgG (H+L)-Alexa Fluor^®^ 488 (Thermo Fisher Scientific, NJ, USA). Negative controls with secondary antibodies were performed. Images were acquired using a fluorescence microscope Zeiss AxioVert 200M. The three-dimensional images (Z-stack) were generated on a Spinning Disk Confocal Microscope ZEISS Cell Observer SD (Peabody, MA, USA). Mean fluorescence intensity was measured using Zen Lite Blue software (Carl Zeiss). For this quantification, the background was initially subtracted, and the fluorescence of the entire field corresponding to the tissue was analyzed. The demarcation of the region was performed using the software’s freehand selection tool to calculate the average fluorescence intensity in the presence of different treatments. The software calculated these results based on the intensity of the corresponding pixels. Four fields per coverslip were evaluated with four animals per group.

### 2.9 Statistical analysis

Results were expressed as mean ± standard error of the mean. Statistical analysis was performed using the t-test or one-way analysis of variance followed by Tukey multiple range tests, using the Prism 8.0.1 software (GraphPad Software, La Jolla, CA, USA). Differences between groups were considered statistically significant when p < 0.05.

## 3 Results

**Figure 1:**
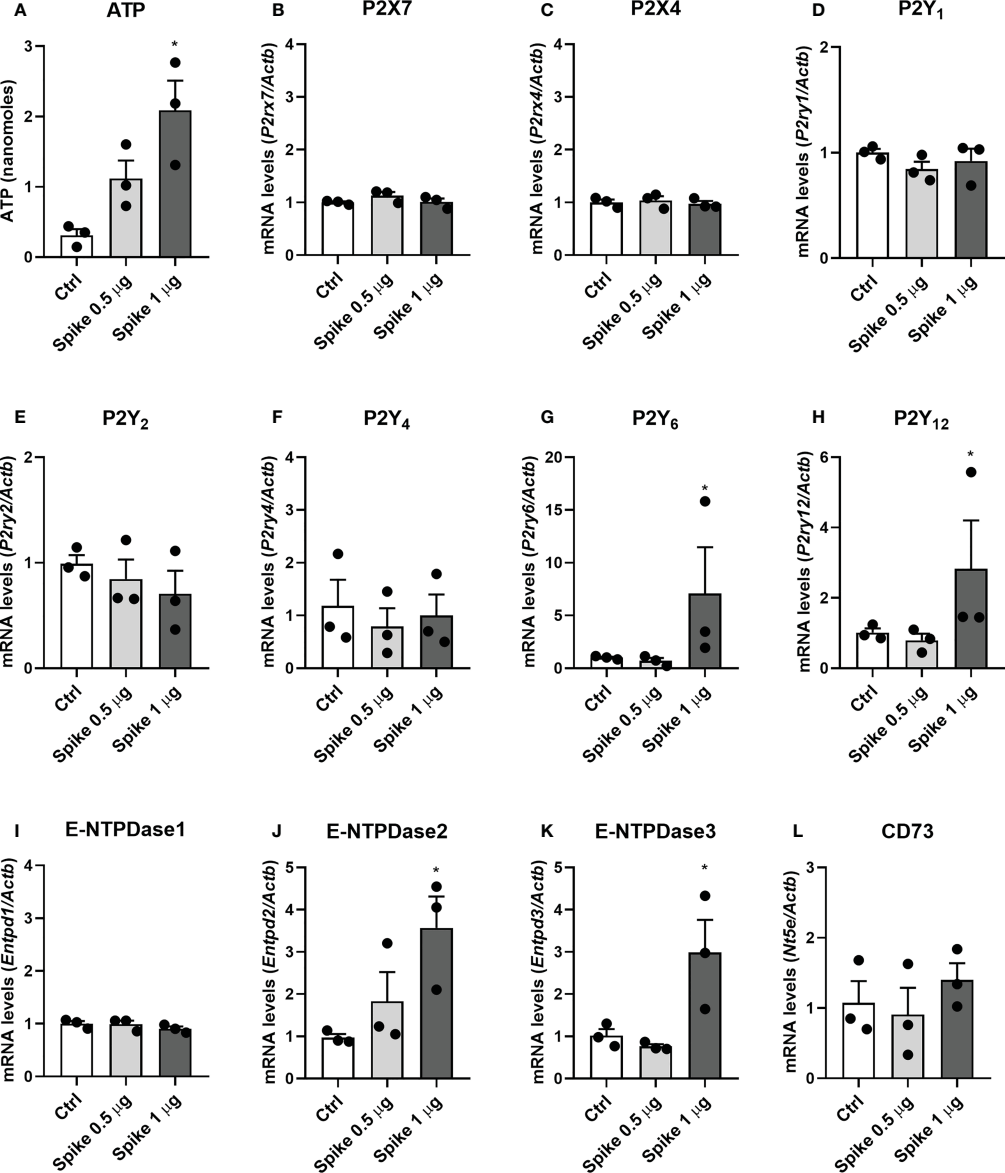
SARS−CoV−2 spike protein increases ATP secretion and purinergic signaling components transcript levels in microglial cells. BV-2 cells were left untreated (Ctrl) or stimulated with 0.5 or 1 µg/mL SARS−CoV−2 spike protein for 2h for ATP quantification in culture supernatants or 24 h for qPCRs. **(A)** ATP concentration in cell supernatants. The levels of transcripts for **(B)** P2X7, **(C)** P2X4, **(D)** P2Y_1_, **(E)** P2Y_2_, **(F)** P2Y_4_, **(G)** P2Y_6_, **(H)** P2Y_12_, **(I)** E-NTPDase1, **(J)** E-NTPDase2, **(K)** E-NTPDase3, and **(L)** CD73 were analyzed by RT-qPCR. Data are representative of three independent experiments (n=3) performed in triplicates and expressed as mean ± SEM. Statistically significant differences between Ctrl and treated groups are represented by asterisks (*, p < 0.05). One-way analysis of variance, Tukey’s test.

**Figure 2:**
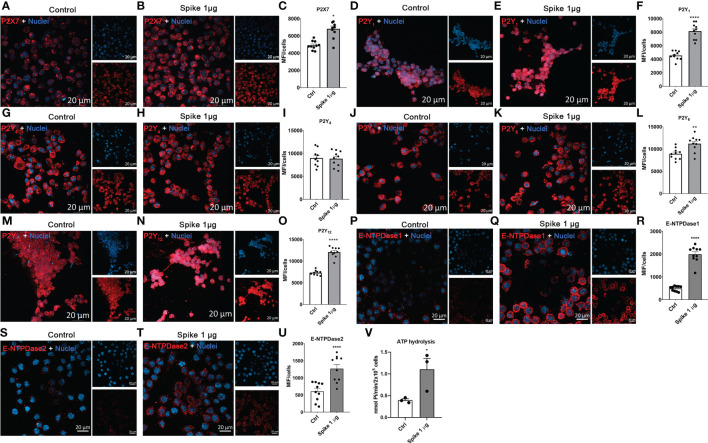
SARS−CoV−2 spike protein increases the expression of P2 receptors and ectonucleotidases in microglial cells. BV-2 cells were left untreated (Ctrl) or stimulated with 1 µg/mL SARS−CoV−2 spike protein for 24 h. Representative images and quantitative analysis for **(A–C)** P2X7, **(D–F)** P2Y_1_, **(G–I)** P2Y_4_, **(J–L)** P2Y_6_, **(M–O)** P2Y_12_, E-NTPDase1 **(P–R)**, and E-NTPDase2 **(S–U)**. Data are representative of three independent experiments (n=3) and expressed as mean ± SEM of 10 fields per condition. **(V)** ATP hydrolysis assay. Data are representative of three independent experiments (n=3) and expressed as mean ± SEM. Statistically significant differences between Ctrl and treated groups are represented by asterisks (*p < 0.05; **p < 0.01; ****p < 0.0001). Student’s t-test. Scale bars: 20 μm.

Extracellular ATP and its derivatives (i.e., ADP and adenosine) have been shown to modulate microglial cell activation and function through purinergic receptors. We observed that stimulation of BV-2 cells with SARS-CoV-2 Spike protein (1 µg/mL) induced ATP secretion (**Figure 1A**) and increased *P2ry6*, *P2ry12*, *Entpd2*, and *Entpd3* transcript levels (p < 0.05; **Figures 1G, H, J, K**, respectively). No significant differences were observed for *P2rx7*, *P2rx4*, *P2ry1*, *P2ry2*, *Entpd1*, and *Nt5e* transcript levels (p > 0.05; **Figures 1B–F, I, L**, respectively) in cells treated with Spike protein at the time point evaluated. No significant changes were detected after 0.5 µg/mL Spike protein treatment (p > 0.05; **Figure 1**). Immunocytochemistry analysis showed that the presence of Spike protein (1 µg/mL) increased the protein expression of P2X7 (p < 0.05; **Figures 2A–C**), P2Y_1_ (p < 0.0001; **Figures 2D–F**), P2Y_6_ (p < 0.01; **Figures 2J–L**), and P2Y_12_ (p < 0.0001; **Figures 2M–O**) receptors in BV2 microglial cells. No differences were observed for P2Y_4_ (p>0,05; **Figures 2G–I**). These data suggest a crucial role of purinergic signaling through diphosphonucleosides (as ADP and UDP) in SARS-CoV-2 infection.

Ectonucleotidases are critical enzymes that control the availability of purinergic receptors ligands. We observed that BV-2 cells stimulated with Spike protein (1 µg/mL) increased E-NTPDase-1/CD39 (p < 0.0001; **Figures 2P–R**) and E-NTPDase2 (p < 0.0001; **Figures 2S–U**) membrane protein expression. In addition, ectonucleotidase assay showed an increased ATP hydrolysis in these cells stimulated with Spike protein (p < 0.05; **Figure 2V**).

**Figure 3:**
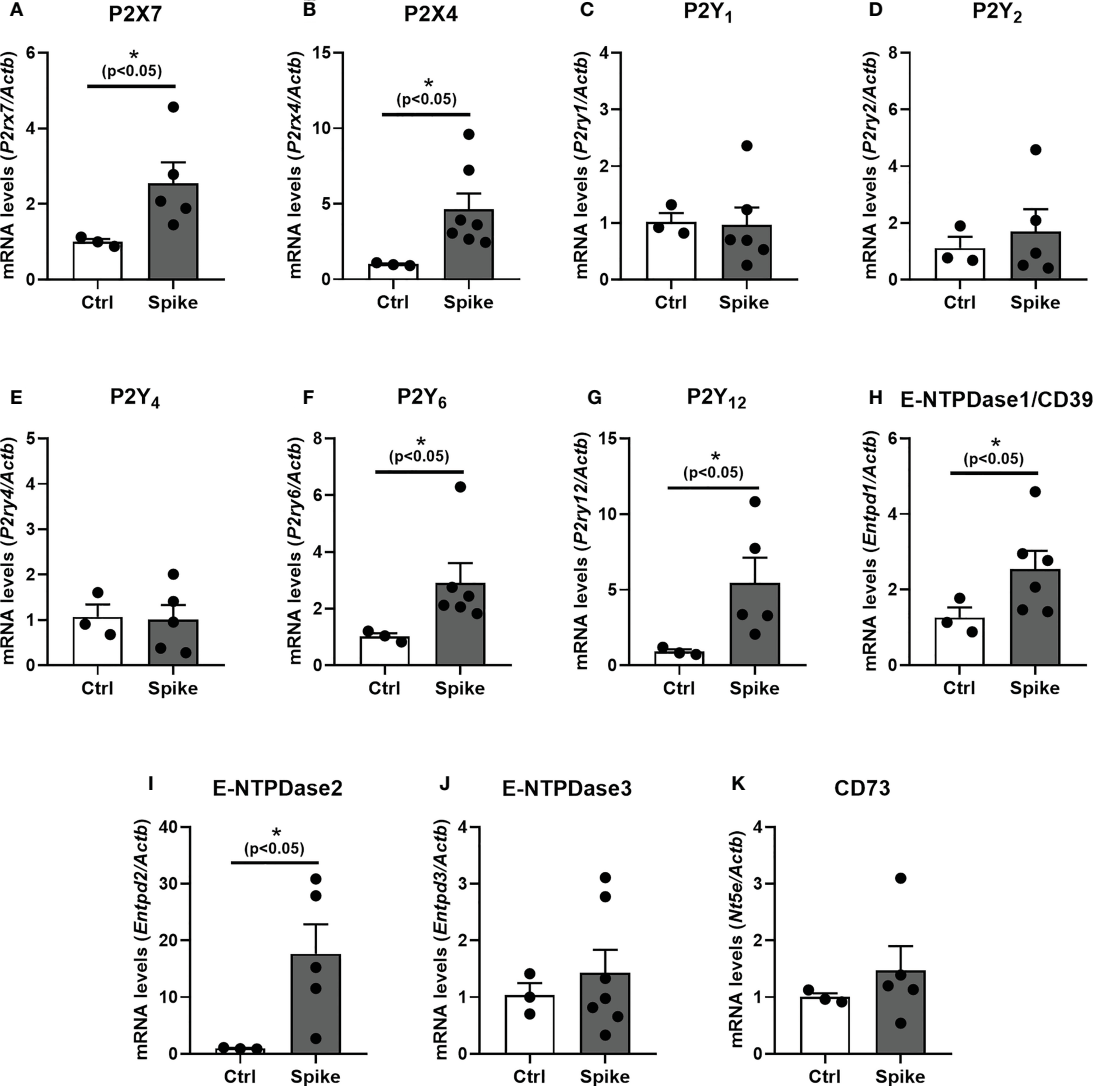
SARS−CoV−2 spike protein increases purinergic signaling components transcript levels in mice hippocampus. The levels of transcripts for **(A)** P2X7, **(B)** P2X4, **(C)** P2Y_1_, **(D)** P2Y_2_, **(E)** P2Y_4_, **(F)** P2Y_6_, **(G)** P2Y_12_, **(H)** E-NTPDase1, **(I)** E-NTPDase2, **(J)** E-NTPDase3, and **(K)** CD73 in hippocampus of mice post-spike ICV injection were analyzed by RT-qPCR. Data are expressed as mean ± SEM. Statistically significant differences between the control (Ctrl) and treated group are represented by asterisks (*, p < 0.05). Outliers were identified and excluded using Prism 8.0.1 software (GraphPad Software, La Jolla, CA, USA). Student’s t-test (Control group n = 3; Spike group n = 5–7).

Using hippocampal tissue of Spike-infused mice (6.5 µg/site, ICV), we found an increase in mRNA levels of *P2rx7*, *P2rx4*, *P2ry6*, and *P2ry12* receptors (**Figures 3A, B, F, G**, respectively; p < 0.05). Likewise, we also found and up-regulation of ectonucleotidases *Entpd1* and *Entpd2* in the hippocampus of mice hippocampus after Spike brain infusion (**Figures 3H, I**; p < 0.05). No significant differences were observed for *P2ry1*, *P2ry2*, *P2ry4*, *Entpd3*, and *Nt5e* transcripts (p > 0.05; **Figures 3C, D, E, J, K**, respectively).

**Figure 4:**
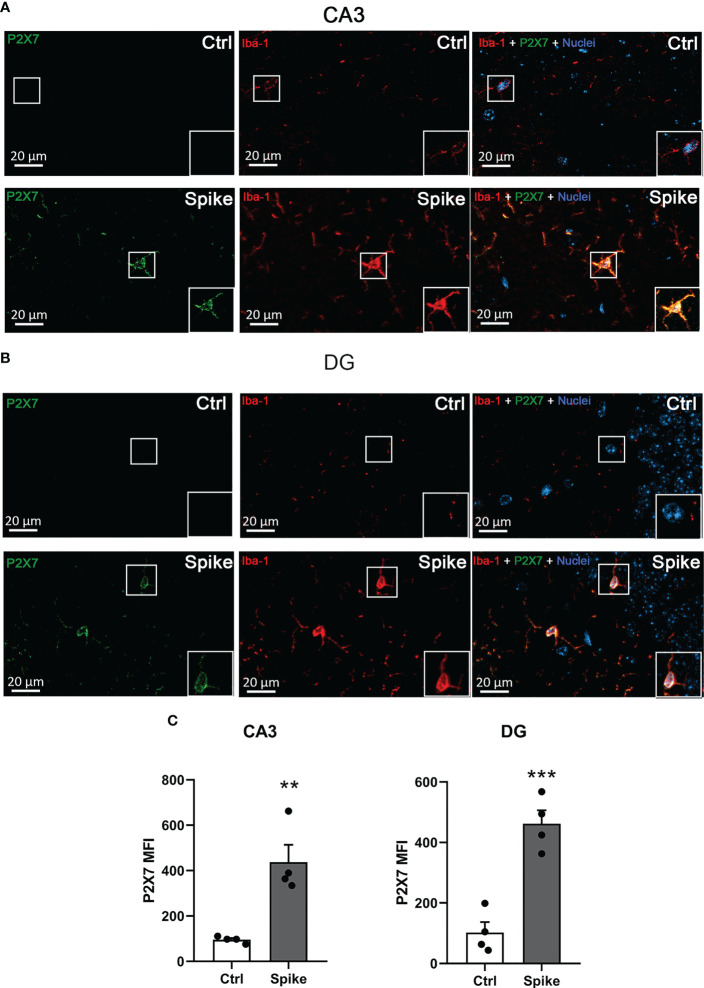
SARS−CoV−2 spike protein increases P2X7 receptor expression in hippocampal microglial cells. Representative images of P2X7 receptor and Iba-1 immunostaining in the CA3 **(A)** and dentate gyrus (DG) **(B)** hippocampal regions of Ctrl or spike-infused mice. Scale bar = 20 μm. **(C)** Quantification of P2X7 receptor fluorescence in Iba-1-positive cells from control (Ctrl) or spike-infused mice in CA3 and DG hippocampal regions. Data are expressed as mean ± SEM. Statistically significant differences between Ctrl and treated groups are represented by asterisks (**, p < 0.01; ***, p < 0.005). Student’s t-test (n = 4).

The P2X7 receptor is the most relevant in neuroinflammatory diseases. Our immunofluorescence analysis confirmed an increase in microglial P2X7 expression in CA3 (**Figures 4A, C**; p > 0.01) and DG (**Figures 4B, C**; p < 0.001) hippocampal regions from spike-infused mice, suggesting that ATP-P2X7 signaling might be involved in neurological alterations induced by SARS-CoV-2 infection.

## 4 Discussion

The emergence of SARS-CoV-2 triggered substantial efforts to understand its pathophysiology and long-term sequelae. Among COVID-19 manifestations, one of the most worrying is the development of multiple neurological complications (8, 26). Microglia are vital cells for inflammatory responses against central nervous system-invading pathogens. We and others previously found that SARS-CoV-2 spike protein induced microglia activation and cytokine secretion *via* TLR4 receptor activation (10, 13). In addition to TLRs, purinergic receptors modulate microglia activation and migration (18) and are critical for the host’s defense against infections (15, 27, 28). This study provides the first experimental evidence that SARS-CoV-2 spike protein induces ATP secretion and alters purinergic receptors and nucleotide metabolizing enzymes *in vitro* and *in vivo* in microglial cells.

Among the P2 receptors, P2X and P2Y_11_ receptors are chiefly activated by extracellular ATP; ATP and UTP activate P2Y_2_; while ADP activates P2Y_1_, P2Y_12_, and P2Y_13_. P2Y_4_, P2Y_6_, and P2Y_14_ are activated by uridine nucleotides UTP, UDP, and UDP-glucose, respectively (14, 16). Of note, the rat and mouse P2Y_2_ and P2Y_4_ are equipotently activated by ATP and UTP, and P2Y_11_ has not been cloned in rodents (29, 30). P2X7, P2X4, P2Y_1_, P2Y_4_, P2Y_6_, and P2Y_12_, are the central P2 receptors that modulate microglial function in health and disease. Even though P2X4 and P2X7 are similar in their structure, P2X4 is highly sensitive and can be sensitized by low ATP concentrations (16).

P2X4 and P2X7 receptors are ion channels upregulated in neuroinflammatory conditions (17, 31, 32). These receptors induce the production of free radicals and increase oxidative stress in inflammatory and neurodegenerative diseases (33, 34). TLR4 stimulation increases P2X7 expression in microglial cells and astrocytes (35–37). P2X7 receptor inhibition decreases LPS-induced cytokine production in cultured human microglial cells and brain tissue of septic mice (33, 36). Notably, the P2X7 receptor is the second signal for NLRP3 inflammasome activation and IL-1β secretion, a cytokine intimately related to synaptic loss and depressive-like behavior following inflammatory and chronic stress conditions (38–40).

Therefore, the increase in P2X7 receptor levels in BV2 microglial cells and the hippocampus of mice injected with SARS-CoV-2 spike protein suggests that P2X7 might contribute to neuroinflammatory events related to COVID-19. Indeed, the P2X7 receptor is involved in microglial-related neurological conditions, including Alzheimer’s disease, HIV-related dementia, and sepsis-associated encephalopathy (41–43). In Alzheimer’s disease, the characteristic accumulation of β-amyloid peptide may induce neuroinflammation through P2X7 receptor mobilization and subsequent activation of NLRP3/caspase-1 pathway (42). Furthermore, the aggregation of these peptides, associated with stimulation by LPS, activates microglia to an M1 proinflammatory profile characterized by TLR4/MyD88/nuclear factor-kappa B signaling pathway activation and cytokine release. Pharmacological or genetic ablation of the P2X7 receptor shifts microglial cells toward an anti-inflammatory and neuroprotective M2 profile, with a reduction in IL-1β production and better cognitive outcomes (42). Interestingly, the HIV glycoprotein gp120 also increases P2X7 receptor protein and mRNA levels in BV2 microglial cells, contributing to nuclear factor-kappa B activation and secretion of inflammatory molecules, leading to microglial loss and neurological alterations such as memory loss and cognitive impairment (41, 44).

Our analysis revealed an increased expression of P2Y_1_, P2Y_6_, and P2Y_12_ receptors (in addition to the P2X7 receptor) in BV2 cells and mice hippocampus. The P2Y_12_ receptor is deeply involved in microglia motility (18, 45, 46). P2Y_12_ expression and levels of inducible nitric oxide synthase (iNOS) increase in mouse brain following nitroglycerin treatment in the chronic migraine murine model. Furthermore, inhibition of the P2Y_12_ receptor reduced iNOS expression in mouse medulla and decreased iNOS, IL-1β, and TNF-α concentrations in BV2 cells pretreated with LPS (47).

The P2Y_6_ receptor plays a crucial role in microglia after brain injury, promoting the clearance of damaged cells and dangerous debris by stimulating phagocytosis. This phenomenon occurs primarily in brain lesions caused by an ischemic stroke, accelerating the recovery of the damaged area (48, 49). Nonetheless, this phagocytic process can be unspecific and culminates in destroying viable cells. Indeed, knockout of the P2Y_6_ microglial receptor inhibits phagocytosis of these intact cells, preventing neuronal loss and memory difficulties in two models of neurodegeneration linked to Alzheimer’s disease, related to β-amyloid peptides or tau proteins (50). Inhibition of P2Y_6_/UDP signaling using exogenous apyrases, reactive Blue 2 (a general P2 inhibitor), or a P2Y_6_ receptor-specific inhibitor prevents neuronal loss after LPS stimulation (51). LPS treatment coupled to a purinergic ligand (2-methylthioladenosine-5′-diphosphate; 2MeSADP) increased the expression of ionized calcium-binding adapter molecule 1 (Iba-1), a marker of microglia activation, and increased P2Y_6_ receptor, CD11b and DAP12 expression, which are proteins involved in microglial phagocytosis (52). Indeed, LPS stimulation induces UDP secretion in BV2 cells, increasing the production of inflammatory cytokines. The knockdown of the P2Y_6_ receptor improves neuronal cell viability and diminishes apoptosis in cells (SH-SY5Y, N2a, and PC12 cells) exposed to a conditioned medium from LPS-stimulated BV-2 cells. These findings suggest that these P2Y receptors might be involved in neuroinflammatory and neurological consequences of SARS-CoV-2 infection.

Ectonucleotidases are critical enzymes that regulate the composition and magnitude of purinergic signaling by controlling extracellular nucleotide levels. These enzymes differ in their functionality and substrate preferences. E-NTPDase1/CD39 has catalytic properties for the hydrolysis of ATP and ADP at the same ratio (1:1). In contrast, E-NTPDase2 and E-NTPDase3 prefer to hydrolyze ATP over ADP in a proportion higher than 30-fold and 3-fold, respectively (53, 54). In the current work, we found that the relative expression of the enzymes that prefer to hydrolyze triphosphate nucleotides (i.e., E-NTPDase2 and E-NTPDase3) were upregulated after spike protein stimulation *in vitro*, probably favoring ADP/UDP generation and (therefore) a diphosphate nucleotide signaling pathway by activating P2Y_1_, P2Y_6_, and P2Y_12_ receptors. *In vivo*, E-NTPDase1 and 2 were also upregulated, agreeing with our *in vitro* data.

Although these P2Y receptors are related to neuroinflammation, the rise of ADP production could be correlated with a mechanism to prevent the disease progression because infected neurons secrete nucleotides that activate purinergic receptors in microglia to digest and eliminate compromised cells, as seen in herpes simplex virus-1 encephalitis (55); these authors observed increased E-NTPDase 1 activity which degrades ATP into ADP and activates the P2Y_12_ receptor in microglia, extending and migrating to damaged sites and eliminating virus-infected neurons. Future studies with knockout animals and specific inhibitors for these P2 receptors and ectonucleotidases are required to understand their role in SARS-CoV-2 infection. Further studies are also required to evaluate purinergic signaling in other cell types using different Spike concentrations or the active virus because the amount of circulating Spike during the course of infection, as well as the extent of its persistence in the blood, varies considerably among COVID patients (56, 57), making it difficult to replicate it in cell cultures. Nevertheless, the concentrations of Spike protein used in our *in vitro* and *in vivo* studies were in the same order of magnitude used in previous studies (8, 10, 58, 59).

In summary, this study provides the first evidence that SARS-CoV-2 infection alters purinergic signaling. SARS-CoV-2 spike protein upregulates purinergic receptors involved in neuroinflammatory and neurodegenerative diseases (i.e., P2X7, P2X4, P2Y_6_, and P2Y_12_). In addition, spike protein increased the expression of E-NTPDase1 and 2, which favors the formation of diphosphate nucleotides and, consequently, the activation of P2Y_6_ and P2Y_12_ receptors. Our findings suggest that these receptors have substantial potential to serve as therapeutic targets to treat or limit neurological changes and cognitive impairments following SARS-CoV-2 infection. Such findings open new avenues for future studies evaluating the specific roles of purinergic receptors in SARS-CoV-2 infection.

## Data availability statement

The raw data supporting the conclusions of this article will be made available by the authors, without undue reservation.

## Ethics statement

The animal study was reviewed and approved by Institutional Animal Care and Use Committee of the Federal University of Rio de Janeiro (protocol number: 068/2).

## Author contributions

LS made substantial contributions to conception and design, data collection, analysis, and interpretation. LS and RL-A drafted the manuscript. VA and SS participated in material preparation, data collection, and analysis. EP-P, RR, MC, GF, LA, EL, and FF-D participated in material preparation and data collection. EK, JS, FF-D, GP, CF, and RC-S contributed to the conception, design and interpretation of data, and manuscript preparation. All authors contributed to the article and approved the submitted version.
